# Infective Endocarditis

**Published:** 1903-11-28

**Authors:** Arthur Maude


### Hospital Clinics and Medical Progress.

### INFECTIVE ENDOCARDITIS.

By Arthur Maude, M.R.C.S.Eng., L.R.C.P.

(*Concluded from page* 129.)

Our ideas as to the duration of infective endocarditis are undergoing change. Twenty years ago any person assumed to be the subject of malignant endocarditis was regarded as doomed within a few weeks. Though we must still regard infection of the cardiac valves as a most formidable condition, there is still no doubt that patients do survive even an extreme destruction of valve-tissue, and that the valves may make considerable attempts at repair. "Changes in a conservative direction may go on when the disease is much prolonged"—so Osler[6] wrote nearly 20 years ago; it behoves us therefore to realise how chronic some cases are. Of Ware's 25 cases, eight were in hospital six weeks or longer. Those cases brought forward by Dr. W. Ewart[7] and Mr. A. S. Morley show that a duration of nine months to a year can be attained. Green[8] recorded cases of six and 18 months' duration. A very prolonged case of Dr. Sydney Coupland's[9] manifested a continued irregular pyrexia of remittent type from November 19th to the following March, when death occurred.

The importance of Dr. Ware's paper lies in the highly critical attempt he has made to distinguish between the varieties of infective organisms which might be present from the differences in clinical behaviour. The cases at his command are too few to afford great results, but he has made certain broad deductions. Not much can be deduced from the temperature-charts, except in the case of pneumonic infection with pneumonia of the lung. It is clear that it is the pneumonia of the lung which produces the pneumonic type of fever. The cases of pneumonic endocarditis without pneumonia show an intermittent form of chart. This is the commonest form of chart, but it cannot be claimed to indicate any particular variety of infective organism, but merely a chronicity of action. Hæmorrhages and rashes, which are apt to co-exist, are, if we accept Dr. Ware's figures, unquestionably more frequent when streptococci are present.

The existence of diarrhœa points to no special organism, but is indicative of a higher range of temperature. Metastatic abscesses are more liable to occur from infection by staphylococci than streptococci.

The cases in which the pneumococcus is present are of profound interest, for they must be examined from the clinical standpoint of pneumonia in which endocarditis occurs. In fatal cases of pneumonia endocarditis has been found in a high proportion—in at least 4 per cent. of Wells's cases and in 16 per cent. of Osler's. Wells[10] noted various important points in pneumonic endocarditis; the brunt of attack usually falls on the left side of the heart, not on the right, as some authorities have asserted. The endocardium in pneumonia is very prone to ulcerate, though ulceration may occur early or may be delayed for weeks. The endocarditis may occur very early, and has been found in cases fatal on the seventh and ninth days (Wells). The course of the fever in the pneumococcus cases given by Ware is highly instructive. In one instance, where the pneumococcus alone was isolated, the fever was of the continuous type seen in pneumonia of the lung, and pneumonia of the lung was present. The other pure pneumococcus case ran a chronic temperature of hectic form, and though the pneumococcus was cultivated from the cardiac valves there was no pneumonia of the lung. Another chart began as a fever of pneumonia, and apical pneumonia was recognised; crisis occurred on the eleventh day, but ten days after the temperature ran up, and the patient died three days later without having had any physical signs of cardiac disease. He had, however, an embolism of the right coronary artery and a large vegetation on an aortic valve. Both diplococcus pneumoniæ and staphylococcus aureus were isolated. The infection with the latter organism clearly only took place a week at least after the lung had begun to resolve. According to Wells, "a not uncommon sequence is for the pneumonia to run its course to crisis with nothing to call attention to the heart. Then, after a few days of apparent convalescence, signs of embolism with fever supervene."

Among the many interesting problems of bacteriology there are few of higher clinical importance than that of "agglutinative reactions." It is impossible within the limits of this paper to discuss the conditions that may produce Widal's reaction, which are yet not enteric. One of the most striking examples of these exceptions was produced by Dr. Hale White before the Clinical Society in 1902.[11] A patient was admitted under suspicion of typhoid, but no agglutination reaction could at first be obtained. Later on the signs and symptoms of infective endocarditis were recognised; the blood afforded pure cultivations of streptococcus longus, but no typhoid bacilli. At the same time Widal's test was tried again and again with different dilutions, but even 1 in 200 gave the reaction. The post-mortem investigation yielded no evidence of typhoid ever having existed.

Dr. Hale White's patient was treated with various strains of antistreptococcus serum, but with no good result. We can scarcely say that the treatment by mixed antistreptococcus serum is satisfactory even in such instances as indicate a pure streptococcus invasion. But even where inoculation does not secure recovery it often tends to improvement sufficiently marked to lead those in charge to believe that its earlier employment might have afforded success. Drs. Cooper and Ogle[12] have recorded such an example; the case afforded cultivations of streptococcus pyogenes, and was treated by a serum from six mixed human infections, prepared by Messrs. Burroughs and Wellcome.

We are in a position already to formulate certain rules as to the use of antistreptococcus serum: (1) No good will result if some other potent organism, as the gonococcus or pneumococcus are present either alone or in combination with streptococci; and (2) that the inoculation must be made early. Another form of treatment recently suggested is daily inunction with a 20 per cent. ointment of protargol; while Dr. Ewart has proposed the intravenous injection of antiseptic agents.

There has been a growing tendency of late to attempt the administration of various antitoxic serums by other means than by subcutaneous injection. Sir Dyce Duckworth [13] reports a most interesting instance in which mixed antistreptococcus serum was administered by rectum for infective endocarditis. Ten c.c. were given daily for nearly six weeks, with excellent results.

[6] Gulstonian Lectures, 1888. [7] Trans. Medico-Chirurg. Soc., 1902. [8] Lancet, vol. i., 1884, p. 800. [9] Brit. Med. Jour., 1885, i., p. 593. [10] Jour. of Amer. Med., Oct. 18, 1902, p. 987. [11] Brit. Med. Jour., 1902, i., p. 715. [12] Ibid., i., p. 1138. [13] Ibid., 1903, i., p. 1195.

